# Megataxonomy and global ecology of the virosphere

**DOI:** 10.1093/ismejo/wrad042

**Published:** 2024-01-10

**Authors:** Eugene V Koonin, Jens H Kuhn, Valerian V Dolja, Mart Krupovic

**Affiliations:** National Center for Biotechnology Information, National Library of Medicine, National Institutes of Health, Bethesda, MD 20894, United States; Integrated Research Facility at Fort Detrick, Division of Clinical Research, National Institute of Allergy and Infectious Diseases, National Institutes of Health, Fort Detrick, Frederick, MD 21702, United States; Department of Botany and Plant Pathology, Oregon State University, Corvallis, OR 97331, United States; Institut Pasteur, Université Paris Cité, Archaeal Virology Unit, 75015 Paris, France

**Keywords:** virus taxonomy, virus evolution, virus host range, virus ecology, metagenomics, metatranscriptomics

## Abstract

Nearly all organisms are hosts to multiple viruses that collectively appear to be the most abundant biological entities in the biosphere. With recent advances in metagenomics and metatranscriptomics, the known diversity of viruses substantially expanded. Comparative analysis of these viruses using advanced computational methods culminated in the reconstruction of the evolution of major groups of viruses and enabled the construction of a virus megataxonomy, which has been formally adopted by the International Committee on Taxonomy of Viruses. This comprehensive taxonomy consists of six virus realms, which are aspired to be monophyletic and assembled based on the conservation of hallmark proteins involved in capsid structure formation or genome replication. The viruses in different major taxa substantially differ in host range and accordingly in ecological niches. In this review article, we outline the latest developments in virus megataxonomy and the recent discoveries that will likely lead to reassessment of some major taxa, in particular, split of three of the current six realms into two or more independent realms. We then discuss the correspondence between virus taxonomy and the distribution of viruses among hosts and ecological niches, as well as the abundance of viruses versus cells in different habitats. The distribution of viruses across environments appears to be primarily determined by the host ranges, i.e. the virome is shaped by the composition of the biome in a given habitat, which itself is affected by abiotic factors.

## Introduction

Most organisms are hosts to multiple viruses and other mobile genetic elements (MGEs) that are parasites, commensals, or mutualists with respect to their hosts [1-5]. Theoretical models suggest that emergence of selfish genetic elements is an intrinsic feature of replicator systems and that such elements were associated with cells from the earliest stages of the evolution of life and accompanied life forms throughout their subsequent diversification over ≈3.5 billion years [6-9]. Viruses and other MGEs have enormously diverse genome lengths (from several hundred nucleotides to more than 2 megabases) and gene compositions as well as distinct replication and expression mechanisms even though hosts may be closely related or shared [3]. In contrast to cellular life forms, which all have double-stranded (ds) DNA genomes, virus genomes (i.e. the nucleic acid molecules that are packaged into virions) are composed of all forms of RNA and DNA [10].

During the last decade, virus diversity research underwent a revolutionary transformation primarily due to advances in metagenomics and metatranscriptomics enabling a broad, high-throughput characterization of, respectively, DNA and RNA viromes from a wide variety of environmental and host-associated habitats [2, 11-21]. Consequently, the known variety of viruses expanded by orders of magnitude, and metagenomics and metatranscriptomics have become by far the most important source of new virus discovery. Recognizing this new reality, the International Committee on Taxonomy of Viruses (ICTV) adopted Code rules for official virus taxon establishment solely on the basis of genome sequence analysis [12].

The advances of metagenomics and metatranscriptomics motivated efforts on comprehensive exploration of the evolutionary relationships among viruses and development of a universal taxonomic system for viruses that included a megataxonomy, i.e. high-ranked taxa such as realms, kingdoms, and phyla [3, 22]. The current megataxonomy is based on shared, broadly conserved virus hallmark genes (VHGs) that encode key components of virions and/or the virus replication machineries incorporating the great majority of known viruses, and is being updated continuously as both culture-dependent and culture-independent efforts result in new virus discoveries [3, 7, 22].

In this review article, we present the megataxonomy of viruses as it was established in 2020–21, followed by discussion of subsequent discoveries that call for local revision and extension of that taxonomic structure. We then use the megataxonomy as the framework to discuss host ranges, ecological distributions, and global and local abundances of major groups of viruses.

### Megataxonomy of viruses

Taxonomy is the classification of biological entities based on perceived evolutionary relationships among them. Recently formally reaffirmed by the ICTV, taxonomy of viruses is no exception to this general principle [23]. Apart from taxonomy, a variety of other classifications are possible and some of these remain useful and important. For example, classification of viruses by the range of their hosts is of practical importance in human and veterinary medicine as well as in agriculture, whereas classification by the type of interaction between viruses and with their hosts (parasitic, commensal, or mutualistic) is important for evolutionary theoretical studies. Particularly relevant to virologists is the classification of viruses by their gene expression strategies that depend on the type of their genome. These so-called Baltimore classes (BCs), named after David Baltimore who co-discovered reverse transcription and who pioneered this classification in 1971 [24, 25], reflect some of the evolutionary relationships among viruses, but they are not valid as high-rank taxa [3, 10].

In any legitimate taxonomy, all taxa must be monophyletic (all members must have a single common ancestor) which, in the case of viruses, presents a major problem. Indeed, in sharp contrast to cellular life forms, which all share about 100 universal genes that can be traced to the Last Universal Common Ancestor [26] and thus can be used to construct global phylogenetic trees (the tree of life), there is not a single gene common to all viruses [3]. Therefore, viruses are polyphyletic (have multiple origins) and, accordingly, a unifying phylogeny of all viruses is impossible to obtain. However, large groups of viruses share small sets of genes encoding key components of the virions and/or of the replication machinery, dubbed VHGs. Viruses sharing a particular set of VHGs or even a single VHG are united into realms (the highest rank in virus taxonomy, with no counterpart in organismal taxonomy), which are subdivided into kingdoms, phyla, classes, orders, families, genera, and species, based both on the phylogenies of the VHGs and on the presence of additional shared genes that can be used for resolving relationships at lower ranks [3, 22].

**Figure 1:**
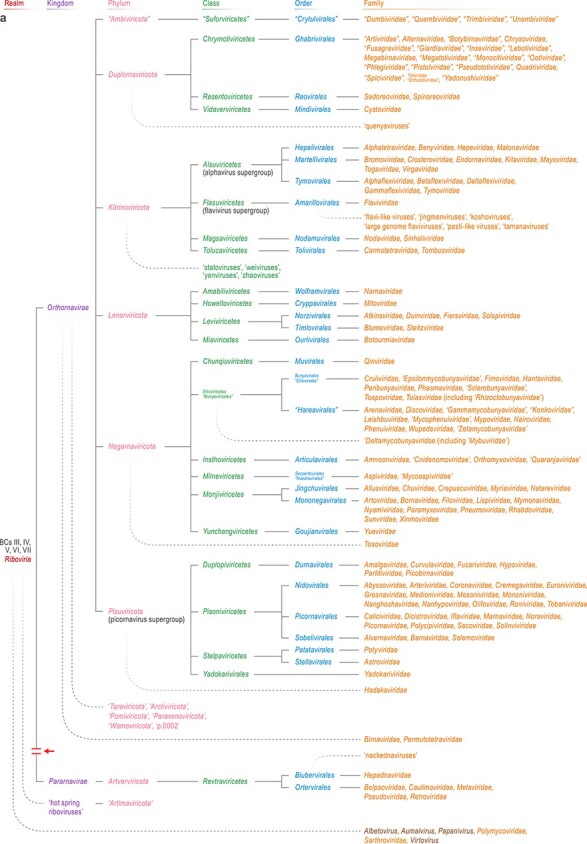

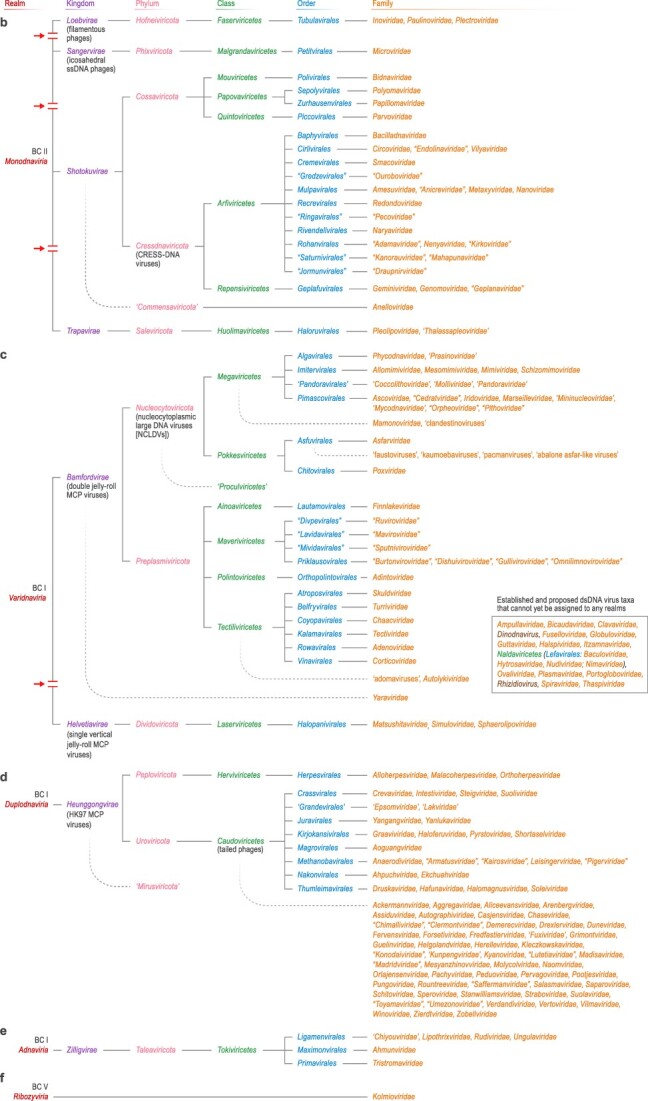
Megataxonomy of viruses and projected split of three of the realms; (A–E) the six ICTV-recognized realms of the current megataxonomy of the virosphere from the highest (realm) rank to the family rank (adapted and amended from [3]); quotation marks around names indicate all above-genus taxa that have been approved by the ICTV Executive Committee in 2023 but have not yet been ratified; apostrophes around names indicate some of the taxa that have been suggested in publications but that have not yet been officially proposed and hence have no official standing; approved but not yet ratified taxon name changes are indicated by vertical juxtaposition of both names; genera (ending with -*virus*) are only indicated if they are ICTV recognized but unassignable to a realm or to a rank below realm; solid lines indicate currently accepted hierarchical relationships among taxa; fading curved dotted lines indicate suspected but uncertain, and hence unofficial, relationships; BCs are superimposed on each realm name to illustrate the incongruency of the Baltimore classification with the current understanding of viral evolutionary relationships. BC I, Baltimore Class I (dsDNA genome); BC II, Baltimore Class II (ssDNA genome); BC III, Baltimore Class III (dsRNA genome); BC IV, Baltimore Class IV (positive-sense ssRNA genome); BC V, Baltimore Class V (negative-sense RNA virus genome including ribozyvirians); BC VI, Baltimore Class VI (positive-sense RNA genomes that are reverse transcribed); BC VII, Baltimore Class VII (dsDNA genomes that are reverse transcribed); (A–C) arrows indicate proposed splits of current realms into separate realms corresponding to the current kingdoms.

Initially, four vast realms of viruses were established (i.e. *Riboviria*, *Monodnaviria*, *Varidnaviria*, and *Duplodnaviria*), each including an enormous diversity of viruses; two much smaller realms were subsequently added. Thus, at the top, the current virus megataxonomy consists of six realms (Fig. 1) [3, 22, 23, 27].

Realm *Riboviria* consists of viruses with positive-sense and negative-sense single-stranded (ss) RNA genomes, double-stranded (ds) RNA genomes, as well as reverse-transcribing viruses with RNA or DNA genomes, i.e. encompasses five BCs (Fig. 1A). The viruses of this realm are unified by homologous RNA-directed RNA polymerases (RdRPs) and reverse transcriptases (RTs). It appears most likely that the common ancestor of this virus realm was a simple virus with a short genome encoding a polymerase that might have been either an RdRP or RT or, perhaps, possessed both RNA-directed activities [28]. The realm splits into two kingdoms: *Orthornavirae* (viruses that encode RdRP and have no DNA stage in their reproduction cycles) and *Pararnavirae* (viruses that encode RTs and replicate by alternating RNA and DNA stages). Because RdRP/RT is the only VHG that is conserved across all ribovirians (see **Glossary** for taxon rank-specific suffixes in vernacular names), all large-scale studies on the evolution and hence taxonomy of these viruses rely on polymerase phylogeny. Phylogenetic analysis of orthornaviraens revealed five distinct branches that were designated phyla [28, 29]. Three of these phyla consist of positive-sense RNA viruses (although one of these phyla, *Pisuviricota*, also includes some groups of dsRNA viruses), one of dsRNA viruses and one of negative-sense RNA viruses. The phyla are strongly supported by phylogenetic analyses although the relationships among them remain largely uncertain and subject to debate [16-18, 30]. However, one feature, the basal position of phylum *Lenarviricota*, is highly robust in RdRP trees rooted by RT. This phylum consists of ribovirians that infect bacteria and their direct descendants from eukaryotes, implying that all the diverse RNA viruses of eukaryotes evolved from bacterial ancestors.

Pararnaviraens are less diverse than orthornaviraens, with only one phylum and class, which splits into two orders, *Ortervirales* and *Blubervirales* [31]*.* Ortervirals share homologous reverse-transcribing replicases (RTs), aspartic proteases, and a set of structural proteins but can have either RNA or DNA genomes that replicate via a DNA or RNA intermediate, respectively. *Blubervirales* includes human hepatitis B virus and its relatives infecting other vertebrate animals. Blubervirals possess DNA genomes that replicate via an RNA intermediate and their structural proteins are unrelated to those of ortervirals [32]. Pararnaviraens are limited in their spread to eukaryotic hosts and apparently evolved from non-viral retroelements, such as Group II self-splicing introns and retrotransposons, by acquiring host proteins and exapting them for structural roles in the virions. Furthermore, the two orders within this kingdom evolved apparently at two independent points of origin from different retrotransposon families [33-35].

Realm *Monodnaviria* consists of ssDNA viruses and small dsDNA viruses (papillomavirids and polyomavirids) that are unified by a single VHG that encodes a distinct endonuclease (or its inactivated derivative) involved in the initiation of genome replication (Fig. 1B). The great majority of monodnavirians have short (2–10 kb) circular genomes that replicate via a rolling-circle mechanism initiated by the hallmark endonuclease [36]. The realm splits into four kingdoms: *Loebvirae* (filamentous prokaryotic viruses), *Sangervirae* (icosahedral prokaryotic viruses), *Shotokuvirae* (eukaryotic ssDNA viruses that produce icosahedral virions), and *Trapavirae* (archaeal viruses that produce pleomorphic virions). The replication initiation endonucleases of monodnavirians are homologous to those involved in the rolling-circle replication of small plasmids and, apparently, monodnavirians of each kingdom evolved from different families of such plasmids [37]. Moreover, within the kingdom *Shotokuvirae* alone, there seem to have been several independent events of virus origin from plasmids. In all such events, the capsid protein genes were captured by the emerging viruses independently from other viruses or from host genes.

Realm *Varidnaviria* consists of an enormous diversity of dsDNA viruses infecting bacteria, archaea, and eukaryotes (Fig. 1C) that typically have an icosahedral capsid and are unified by a single shared VHG, a vertical jelly-roll major capsid protein (MCP) which is, however, replaced by unrelated proteins in some groups, resulting in odd capsid shapes [38-43]. The vast varidnavirian kingdom *Bamfordvirae* consists of viruses that possess a double jelly-roll (DJR) MCP and, in most cases, an additional VHG, a genome packaging ATPase of the FtsK-HerA superfamily. Bamfordviraens include both small icosahedral viruses infecting bacteria and archaea and giant viruses of eukaryotes (phylum *Nucleocytoviricota*) as well as several families of smaller dsDNA viruses of eukaryotes [44]. The much smaller kingdom *Helvetiavirae* includes bacterial and archaeal viruses that encode two MCPs, each containing a single vertical jelly-roll domain.

Realm *Duplodnaviria* includes some of the most abundant viruses on Earth, i.e. the tailed dsDNA bacterial and archaeal viruses (*Caudoviricetes*) [4, 45], the recently discovered “mirusviruses” [46] that appear to be associated with a wide range of unicellular eukaryotic hosts (“*Mirusviricota*”), and animal herpesvirals (Fig. 1D). All these viruses share a distinct morphogenetic module that consists of four VHGs encoding a HK97-fold MCP, a genome packaging ATPase-nuclease (large terminase subunit) that is distinct from the functional counterpart of varidnavirians, a portal protein, and a capsid maturation protease. With “mirusviricots” currently remaining unclassified, this realm has a simple taxonomic structure including a single kingdom and two phyla, *Uroviricota* (caudoviricetes) and *Peploviricota* (herpesvirals). Notwithstanding this uniformity at the top ranks of the taxonomy, the diversity of uroviricots is enormous, resulting in a constant flux of reorganization [45, 47].

The four major realms encompass the vast majority of currently known viruses. Although they emerged independently, they are loosely connected by patchy sharing of other conserved proteins, such as various RNA and DNA helicases (in particular, superfamily 3 helicases), chymotrypsin-like proteases, and movement proteins [3].

More recently, two much smaller realms were formally recognized by the ICTV: *Adnaviria* [27] and *Ribozyviria* [48, 49]. Realm *Adnaviria* (Fig. 1E) consists of rod-shaped and filamentous viruses that infect primarily hyperthermophilic archaea of the phylum *Thermoproteota*. The remarkable feature of these viruses is that their encapsidated genome is a linear dsDNA in an A conformation [27, 50]. Adnavirians lack widespread VHGs but share, among themselves, several conserved genes, including a unique α-helical MCP [51]. The small realm *Ribozyviria* includes a single family, *Kolmioviridae* [52] (Fig. 1F), which encompasses human hepatitis D viruses 1–8 (genus *Deltavirus*) and their relatives discovered in other animals [53-58]. Ribozyvirians are viroid-like circular RNA replicons encoding a nucleocapsid protein (delta antigen). Similar to viroids, these viruses hijack the cellular transcription machinery for their genome replication and depend on other viruses (bluberviral hepatitis B virus in the case of deltaviruses, arenavirids in case of daletviruses, unknown for most others) for the formation of infectious enveloped virions [59, 60].

### What is next for virus taxonomy: impact of evolutionary analyses and new virus discoveries

The comprehensive taxonomy of viruses is a recent development, with the four large realms adopted by the ICTV in 2020, followed by the two small ones a year later. However, even in the short time elapsed since, new developments made it abundantly clear that even at its highest ranks, virus taxonomy is a dynamic project that is far from settling into a final form. Revisions will be coming from at least three major sources: detailed analyses of virus evolution, development of taxonomic frameworks for currently unclassified viruses, and discovery of novel viruses, primarily by mining metagenomes and metatranscriptomes.

To begin with fundamental questions stemming from evolutionary analyses, the study of the evolution of both ribovirians and monodnavirians challenges the central concepts of virus megataxonomy. Indeed, although there is a universal VHG uniting each of these realms, and hence all the viruses within each realm are evolutionarily connected, these realms hardly can be considered monophyletic because neither appears to be traceable to a common ancestral virus [61]. Specifically, there seem to be at least three independent founder events whereby viruses evolved from non-viral MGEs in the case of ribovirians and at least four such events for monodnavirians. Taxonomic decisions remain to be made on whether to split the realms, with the current kingdoms becoming independent realms, or whether to retain the current realms based on shared VHGs. Strict adherence to the monophyly criteria seems to require realm splitting (Fig. 1A and B).

Recent large-scale metatranscriptome studies led to a massive expansion of the diversity of the ribovirians by more than an order of magnitude [16-18]. Because this work was based on the identification of the RdRP, the realm structure is not affected, but several new phyla have been suggested and numerous groups are expected to form new classes or orders (Fig. 1A). Moreover, a group of RNA viruses (“hot spring riboviruses,” “*Artimaviricota*”) was discovered encoding an RdRP that is extremely distant from all known RdRPs of ribovirians and, as suggested by structural comparison, appears to be an intermediate between RdRPs and RTs [62]. Potentially, this group might become another ribovirian kingdom.

Eukaryotic viruses in the family *Anelloviridae* were initially considered to be unrelated to other ssDNA viruses, but recent data suggest that they have evolved from a shotokuviraen ancestor and are likely to be assigned into the realm *Monodnaviria* as a new shotokuviraen phylum, “*Commensaviricota*”, alongside *Cressdnaviricota* and *Cossaviricota* [63] (Fig. 1B).

In-depth analysis of the evolution of the DJR and vertical SJR MCPs from cellular ancestors demonstrated their independent origins in the varidnavirian kingdoms *Bamfordvirae* and *Helvetiavirae*, refuting the previous hypothesis on the fusion of two SJR proteins (as in *Helvetiavirae*) yielding the DJR in the common ancestor of the bamfordviraens [64]. These findings indicate that there is no direct evolutionary connection between the two current varidnavirian kingdoms, suggesting that the split of this realm into two separate ones is imminent (Fig. 1C).

Among the known but currently unclassified viruses, several groups are clear candidates for new small realms. Perhaps the most obvious are viruses of archaea with unusual virion architectures, such as those shaped like spindles, bottles, or droplets, that are not connected to any other viruses through homologous VHGs even though some homologous genes less commonly found in viruses are shared [65-68]. Among bacterial viruses, viruses of the family *Plasmaviridae* appear to be unrelated to other known viruses and could be assigned to a separate new realm [69].

Even more radical novelty was brought about by metatranscriptome mining for viroid-like viruses with ribozyme-containing circular RNA genomes [21, 70]. Several groups of previously unknown viruses with viroid-like genome structure have been discovered, some of which are distant relatives of kolmiovirids, whereas others are not. Clearly, a major expansion of the realm *Ribozyviria* (Fig. 1F) is due, but the taxonomic assignments for the rest of the viroid-like viruses remain less than obvious. In particular, “ambiviruses”, which have by far the largest genomes among known viroid-like viruses (≈5 kb), encode a distinct RdRP without a clear affinity to any group of *Riboviria*. Given the presence of the signature VHG of ribovirians, and more specifically, orthornaviraens, these viruses have been approved to be classified as a new ribovirian phylum, “*Ambiviricota*” [71] (ratification pending; Fig. 1A). However, their circular RNA genomes in which both the positive-sense and the negative-sense strands encompass ribozymes implicated in the processing of replicative intermediates structurally resemble those of ribozyvirians, suggesting chimeric origin of such viruses via recombination of a ribovirian and a viroid [21, 70].

Metagenome mining has led to substantial expansion of other large virus realms, as well. Perhaps most notable is the recent discovery of “mirusviricots”, an expansive group of large viruses with dsDNA genomes [46]. “Mirusviricots” encompass the VHGs for structural proteins characteristic of duplodnavirians, whereas most of the genes encoding the replication and transcription machineries are related to those of the large and giant viruses in the varidnavirian phylum *Nucleocytoviricota*. Under the current formal criteria used in virus megataxonomy, “mirusviricots” fall into realm *Duplodnaviria*, where they are likely to become a new phylum (Fig. 1D), but the discovery of this group seems to reveal a previously unsuspected evolutionary link between the two realms of viruses with dsDNA genomes.

Other discoveries could appear less surprising, but they rapidly fill out and thus expand virus realms from within, exposing their previously unsuspected diversity. A notable example is the caudoviricete order *Crassvirales* of *Duplodnaviria* (Fig. 1D) that was founded by crAssphage, the most abundant human-associated virus that was assembled from human gut metagenomes [72, 73]. At the time of its identification, crAssphage appeared unique, and even VHGs including the gene for the MCP were not detected due to the lack of homologs with sufficient sequence similarity. However, subsequent metagenome mining for related viruses using sensitive methods for sequence analysis resulted in the identification of thousands of related viruses with a distinct gene complement, mostly animal-associated but also some coming from various environments [74-76]. This progress stimulated successful efforts on host identification, cultivation, and structure determination of crassvirals [77-80]. Similarly, the genomes of many other caudoviricetes have been assembled from metagenomes, some giving rise to proposed new families and orders [20, 81-86]. Complementary studies have revealed a wide diversity of varidnavirians, in particular, many groups of viruses with small genomes that have been overlooked not only in the environment but also in the human gut [87-90].

### Virus megataxonomy and host ranges

**Figure 2:**
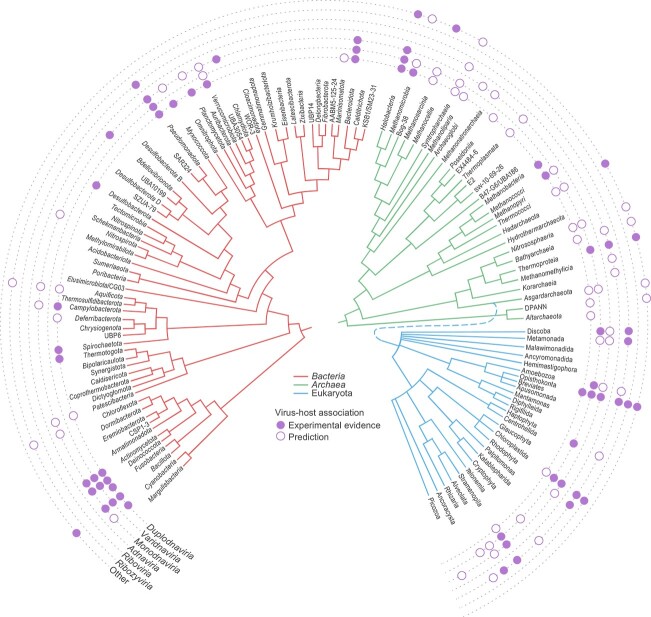
The major patterns of virus–host associations; the figure depicts the distribution of experimentally confirmed and predicted virus–host associations across the three domains of cellular life; schematic phylogenies of bacteria (red) and archaea (green) were obtained from AnnoTree [165], and that for eukaryotes (blue) was adapted from Burki *et al*. [166]; the bacterial and archaeal taxonomy is based on the GTDB database [167]; the experimentally confirmed virus–host associations were retrieved from the Phage & Host Daily database [168] and from previously published data for bacteria and archaea [169], and eukaryotes [5].

Overlaying virus megataxonomy with information on virus hosts reveals a variety of patterns, most of which are difficult to explain (Fig. 2). In this section, we first consider the host ranges of the viruses currently included in the ICTV taxonomy [7] and then discuss changes engendered by mining metagenomes and metatranscriptomes and the possible biological causes of the observed host range patterns. At the top ranks of classification of both viruses and hosts, there are few exclusive associations: viruses of three of the four large realms are represented in each of the domains of life (*Bacteria*, *Archaea*, and Eukaryota), the exception being ribovirians, which have not yet been found in archaea. It remains to be seen if this is a true exclusion of RNA viruses from archaea, or they are missed so far due either to insufficient sampling or to extreme divergence of the presumable RdRP that is unrecognizable by established search methods. However, the abundances of viruses from the different realms as well as the diversity and representation of kingdoms and phyla show major differences among host domains, including many cases of non-overlapping host ranges.

The viromes of prokaryotes (bacteria and archaea) are heavily dominated by dsDNA viruses, primarily duplodnavirians (caudoviricetes), with an apparently more modest contribution of varidnavirians, and in archaea with the addition of adnavirians and several archaea-specific groups that could form new small realms (Fig. 2). However, within realm *Varidnaviria*, the smaller kingdom *Helvetiavirae* (which might merit realm status as discussed above) includes only viruses infecting bacteria and archaea. By contrast, the vast kingdom *Bamfordvirae* combines viruses of prokaryotes and eukaryotes, the latter including the enormously diverse and abundant phylum *Nucleocytoviricota*.

The ssDNA viruses of the realm *Monodnaviria* comprise ≈5% of the known viromes in both bacteria and archaea, and more than 10% of the eukaryotic virome. However, at the kingdom rank, the host ranges do not overlap: two kingdoms are specific to bacteria, one to archaea, and the fourth, largest kingdom, *Shotokuvirae*, is exclusively associated with eukaryotes [37] (Fig. 2).

Among prokaryotes, RNA viruses of the ribovirian kingdom *Orthornavirae* are known to infect only bacteria, where they represent a relatively small fraction of the virome, notwithstanding the recent expansion of bacterial ribovirians [16-18]. In sharp contrast, the virome of eukaryotes is heavily dominated by orthornaviraens. Furthermore, pararnaviraens, the reverse-transcribing viruses, are represented only in eukaryotes, where these viruses comprise ≈5% of the virome diversity. Bacteria and archaea are not known to harbor any reverse-transcribing viruses although they are hosts to diverse non-viral retroelements. In contrast to the bacterial and archaeal viromes, where viruses with dsDNA genome dominate, these viruses account for about 20% of the eukaryotic virome and mostly belong to realm *Varidnaviria*. The two currently recognized small virus realms are host specific. Adnavirians are widespread in archaea but do not appear to be connected to any viruses of bacteria or eukaryotes. The currently classified ribozyvirians are confined to two animal phyla, Arthropoda and Chordata [52], although recent findings in metatranscriptomes suggest a substantial expansion of their host range [21].

At the rank of virus and host phyla, there are many cases of highly specific association of virus taxa with host taxa and, conversely, many exclusions. Thus, of the five established orthornaviraen phyla (Fig. 1A), four are believed to infect (almost) exclusively eukaryotes (but see the discussion of recent metatranscriptome mining results below). The fifth phylum, *Lenarviricota*, consists of the leviviricetes, the largest and rapidly expanding group of bacterial RNA viruses [91, 92], and viruses infecting eukaryotes that appear to have directly evolved from leviviricetes (botourmiavirids [*Miaviricetes*], mitovirids [*Howeltoviricetes*], and narnavirids [*Amabiliviricetes*]). Strikingly, embryophytes (land plants) and fungi are not known to host any viruses with dsDNA genomes (other than the reverse-transcribing caulimovirids in plants), thus excluding two vast viral realms from the plant and fungal viromes. Conversely, plant and fungal viromes are dominated by ribovirians, in particular, in the case of fungi, dsRNA viruses of the rapidly expanding duplornaviricot class *Chrymotiviricetes* and the pisuviricot class *Duplopiviricetes*, although the presence of monodnavians is not negligible either [93-97]. Similarly, one of the two current phyla of realm *Duplodnaviria*, *Peploviricota*, which consists of herpesvirals, is limited to animal hosts [98].

In parallel with the expansion of virus diversity, metagenomic and metatrascriptomic studies often substantially expand the known or predicted host ranges of viruses. Thus, recent metatranscriptome analyses [16-18] led to a disproportionate expansion of the phylum *Lenarviricota*, which currently accounts for ≈30% of the total ribovirian diversity [17]. Furthermore, analysis of metatranscriptomic sequences suggested that some groups of ribovirians, such as durnaviral picobirnavirids that previously were thought to infect animals, are actually bacterial viruses [99, 100], whereas partitivirids apparently include both eukaryotic and bacterial viruses [17, 62]. Moreover, several distinct groups of ribovirians assigned to bacterial hosts based on the genome organization, with the potential to become new phyla or even a kingdom, have been discovered (see above). These recent findings indicate that the contribution of ribovirians to the bacterial virome had been substantially underappreciated even if this virome remains dominated by duplodnavirians. However, three of the five expansive ribovirian phyla, *Negarnaviricota*, *Duplornaviricota*, and *Kitrinoviricota*, that consist of negative-sense RNA viruses, dsRNA viruses, and a broad diversity of eukaryote-infecting positive-sense RNA viruses, respectively, remain conspicuously absent in bacteria.

Metatransriptome mining for viroid-like viruses revealed diverse members of the *Ribozyviria* (Fig. 1F) in numerous data sets that lacked any animal sequences, suggesting that these viruses infect unicellular eukaryotes and thus substantially expanding the host range for this virus realm, although the specific unicellular hosts remain unknown [21]. Similarly, the discovery of “mirusviricots” expands the host range of duplodnavirians to stramenopiles [101] but, considering the genetic diversity of “mirusviricots” [46], the actual host range is likely to be much broader. Undoubtedly, further metagenome and metatranscriptome studies will amend the patterns of host ranges although it appears likely that at the top ranks of the taxonomy for both viruses and hosts, the major trends are already established.

The biological causes of the observed patterns in host ranges are far from being well understood, with only some general considerations appearing relevant. Thus, the wide spread of ribovirians among eukaryotic hosts at the expense of viruses with dsDNA genomes might be linked to the proliferation of intracellular membranes in the eukaryotic cytoplasm that provide a hospitable milieu for RNA virus reproduction [102, 103]. A complementary factor affecting the host range seems to be the nuclear membrane that complicates the access of DNA viruses to the host transcription and replication machineries. Conversely, the current absence of three of the five ribovirian phyla in bacteria might not reflect any intrinsic biological causes of their exclusion, but rather the heavy competition from DNA viruses because of which major diversification of RNA viruses occurred only in eukaryotes.

All these factors likely shape virome composition but certainly do not strictly define virus host range. Along similar lines, the presence or absence of a cell wall as well as its specific structure appears to be an important determinant of the virome composition for different host taxa. In particular, it seems plausible that the enormous spread of caudoviricetes among prokaryotes owes to the function of the tail as a DNA injection device that, together with enzymes such as lysozymes, enables the virus genome to cross the host cell wall [51, 104-106]. Different caudoviricetes have adapted to penetrate either the peptidoglycan-based bacterial cell walls or archaeal S layers. Again, however, this is not a strict requirement for delivering a virus genome; many non-tailed viruses enter bacterial and archaeal cells via other mechanisms, such as internalization following binding to pili (filamentous viruses of monodnavirian kingdom *Loebvirae*, leviviricetes, certain cystovirids, and adnavirians) [107-111] or formation of transient delivery devices (microvirids and tectivirids) [112-115].

The conspicuous absence of viruses with dsDNA genomes from land plants, as opposed to their abundant presence in unicellular green algae, also seems to owe to the inability of viruses to penetrate the robust, cellulose-based wall of plant cells. The systemic spread of plant viruses in the host requires passing through plasmodesmata, which is typically facilitated by dedicated virus movement proteins [116], but size exclusion of plasmodesmal transport seems to prevent the spread of large virions [94]. Along similar lines, viruses do not appear to have evolved means to penetrate the chitin cell walls of fungi, and therefore the known fungal viruses, mostly, orthornavirans and pararnavirans, as well as some monodnavirians, spread either vertically, with the host cell division, or horizontally, via hyphal contact [93].

The notable exceptions discussed above notwithstanding, in general, the host ranges of viruses seem to be mostly shaped not by strict exclusion principles but rather by competition between different types of viruses for organismal and cellular niches. As a result, most of the host range patterns display as differential abundances of virus taxa across the host taxa rather than strictly non-overlapping viromes.

### Ecological patterns across virus megataxonomy

**Figure 3:**
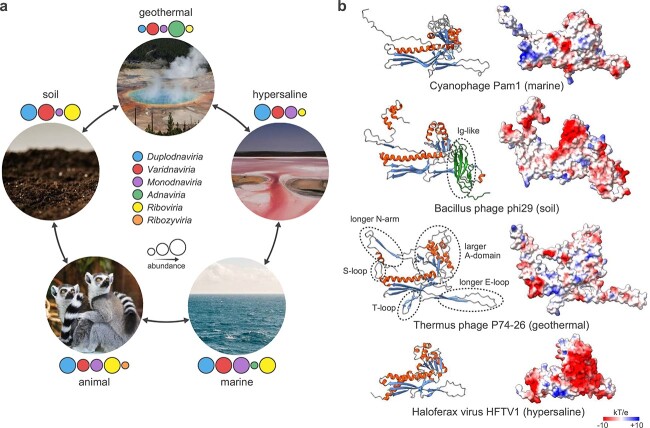
Global ecology of viruses and molecular adaptations to distinct environments; (A) the relationship among environments and viruses of established realms; the photographs representing different environments were downloaded from https://www.pexels.com/; the presence of viruses belonging to different realms is shown with colored circles, with the diameter of the circle roughly representing the abundance of the particular virus realm in the corresponding environment; (B) molecular adaptation of caudoviricetes MCPs to distinct environments; ribbon (left) and electrostatic surface (right) representations of the MCPs of marine cyanophage Pam1 (PDB id: 7EEL), soil Bacillus phage phi29 (PDB id: 6QYD), thermophilic Thermus phage P74-26 (PDB id: 6O3H); in the absence of available high-resolution structures for haloarchaeal tailed viruses, Haloferax virus HFTV1 [170] is represented by an AlphaFold model of the MCP; the immunoglobulin (Ig)-like domain of phi29, a commonly found adaptation in gut viruses, is shown in green and circled; specific adaptations to high-temperature environments, such as longer loops, are circled in the phage P74-26 MCP structure; in the surface representations, the charge distribution from negative to positive is shown using the gradient from red to blue, respectively; in the ribbon representation, α-helices and β-strands are shown in red and blue, respectively.

Metagenomic and metatranscriptomic data provide rich material for exploring associations of different groups of viruses with particular environments that correlate with host ranges but could also depend on abiotic factors. Although the famous microbiological tenet “everything is everywhere but the environment selects” [117] has been questioned for viruses [118], it appears to hold, at least, at the granularity of realms. The picture that emerges from the combination of culture-dependent and culture-independent efforts shows most of the realms being represented across multiple, highly distinct environments (Fig. 3A). Not unexpectedly, extreme environments present the most striking examples. For instance, ribovirians were commonly considered to be excluded from geothermal environments due to instability of RNA at high temperatures. However, a recent study has uncovered two groups of ribovirians apparently infecting hyperthermophilic bacteria in acidic hot springs with temperatures up to 80°C and pH ≈ 2–3 [62] (“hot spring partitiviruses” and “*Artimaviricota*”; Fig. 1A). Similarly, apparent eukaryotic RNA viruses were discovered in hypersaline lakes, an archaea-dominated environment in which RNA viruses were not previously detected [119]. However, whether RNA viruses can infect archaea remains unknown. Conversely, adnavirians infecting hyperthermophilic archaea and previously observed exclusively in terrestrial hot springs have been discovered in marine viromes, where they appear to be associated with *Methanophagales* (ANME-1) [86], a prominent group of methane-oxidizing archaea. Similarly, spindle-shaped viruses that are common in extreme geothermal environments [120, 121] are now known, largely through metagenomics, to infect a wide range of marine mesophilic archaea, including *Asgardarchaeota* [122, 123], *Bathyarchaeia* [124], *Methanophagales* [86], and *Nitrososphaeria* [125], as well as hyperhalophilic [126] and methanogenic [20, 127] archaea.

Members of the three other realms of DNA viruses, namely, *Duplodnaviria*, *Monodnaviria*, and *Varidnaviria*, are even more widely distributed across environments, with representatives of all three realms present in extreme geothermal and hypersaline environments, as well as marine and other moderate ecosystems (Fig. 3A). Here again, metagenomics has been key to gauge the environmental distribution and host associations of these viruses. Filamentous viruses of monodnavirian order *Tubulavirales* present an illustrative example. In a single study, the modest collection of about 60 tubulavirals has been expanded to greater than 10 000 representatives, greatly broadening their host range and demonstrating the presence of these viruses across a wide range of environments [128].

The nearly universal environmental distribution of viruses of all realms appears to reflect both ancestral colonization of the particular biomes by virus–host pairing and host switching, and host range expansion (Fig. 3A). Polyomavirids, small dsDNA viruses of realm *Monodnaviria* (*Papovaviricetes*), present an illustrative example of ecological niche expansion linked to long-term virus–host co-evolution, occasional host switching notwithstanding [129]. Indeed, polyomavirids infect arthropods, fish, birds, and mammals and have been co-evolving with their animal hosts for at least half a billion years [129, 130]. By contrast, analysis of archaeal duplodnavirians revealed recurrent transfers among biomes, including anoxic, hypersaline, and marine environments, apparently largely driven by host switching [45]. For instance, haloarchaeal duplodnavirians of the thumleimaviral *Druskaviridae* family have likely evolved from marine archaeal viruses (magrovirals) [45, 131], with natural mixing across marine and hypersaline ecosystems (e.g. through evaporation of coastal marine water), providing an ecological setting for marine viruses to encounter halophilic archaea.

In eukaryotes, host switching is rather common, especially among more closely related hosts occupying the same ecological niche, with the prime example being zoonoses (diseases caused by viruses transferred to humans from other animals and vice versa [e.g. ribovirian Ebola virus, HIV-1, severe acute respiratory syndrome coronaviruses 1 and 2]) as well as numerous viruses that replicate in both arthropods and mammals (e.g. many orthoflaviviruses). More radical host switches have also occurred over longer phylogenetic distances. For instance, insect and fungal viruses have likely gained the ability to infect plants, thereby changing the ecological niche, by acquiring cell-to-cell movement protein genes enabling transport through plasmodesmata [94, 116, 132].

Adaptation to a new environment typically entails changes in structural proteins because, during the extracellular phase, virions have to cope with new physicochemical and biological challenges. The more radical the environmental transition, the more extensive are the necessary changes. Comparative genomics and structural studies have illuminated some of these molecular adaptations, which we discuss below using duplodnavirians as examples (Fig. 3B). Viruses thriving in extreme geothermal environments need to increase the thermostability of their capsids. Analysis of the Thermus phage P74-26 capsid has shown that its thermostability is ensured through enhanced hydrophobic interactions at the subunit interfaces, which were estimated to be more than 2-fold higher in Thermus phage P74-26 than in homologs from mesophilic viruses [133]. Indeed, the hydrophobic effect increases in strength at high temperatures [134] and has been also implicated in the capsid stability of hyperthermophilic adnavirians, varidnavirians, and spindle-shaped viruses [51, 135, 136]. Longer loops and additional structural elements (Fig. 3B) that play critical roles in intra- or inter-capsomer interactions were identified as additional factors contributing to the thermostability of the Thermus phage P74-26 capsid [133]. Furthermore, the capsid proteins of Thermus phages P74-26 and P23-45 are considerably larger compared to their mesophilic counterparts and, hence, the capsids built from such proteins have larger volumes and can accommodate longer genomes [133, 137]. Thus, it has been hypothesized that decreased icosahedral complexity (smaller triangulation numbers) promotes more stable capsid assembly [133].

Duplodnavirians thriving in hypersaline environments are adapted in various ways, with increased negative charge on the protein surface being the most prominent one (Fig. 3B). The negative surface charge is critical for proper protein folding and stability under conditions in which the salt concentration both inside and outside of the host cell is approaching the saturation point (Fig. 3B). This is a general adaptation to hypersaline environments, typical also of the proteomes of cellular hosts and other viruses. The increased surface negative charge is thought to enable the proteins to compete with ions for water molecules, thereby keeping the proteins in solution [138]. Exposure to low salt conditions reversibly inactivates haloarchaeal duplodnavirians, with infectivity being restored once the virions are returned to the high-salt environment [139], indicating the dependence on, rather than mere tolerance of, high salt concentrations. Enveloped pleolipovirids (realm *Monodnaviria*), such as *Haloarcula hispanica* pleomorphic virus 1, retain high infectivity in saturated salt (5.5 M NaCl) [140].

Enhanced adherence to surfaces appears to be important for viruses found in the gastrointestinal tracts of animals. Many caudoviricetes in the gut encode proteins containing immunoglobulin (Ig)-like domains that are thought to facilitate virion binding either to bacterial cells or the mucosal layer of gut epithelial cells [141-143]. It has been suggested that bacterial virus adherence to mucus was a form of non-host-derived immunity against virus-susceptible bacteria [141]. The same adaptation has been found in archaeal duplodnavirians and varidnavirians infecting gut methanogens [20]. Although Ig-like domains are not exclusive to gut viruses, comparison of viruses associated with the environmental and host-associated methanogens revealed significant enrichment of such domains in the gut [20]. The Ig-like domains can be fused to a range of structural proteins, including MCPs (Fig. 3B), major tail proteins, baseplates, tail fibers, or tail spikes [20, 144]. In some viruses, the Ig-like domains are encoded as stand-alone proteins that decorate the capsid surface. For example, in the iconic Escherichia phage T4, the Hoc protein contains three tandem Ig-like domains and forms elongated fibers that attach to the center of the hexameric MCP capsomers [145].

Except for ribozyvirians, viruses from all current realms are globally distributed, known to thrive in both moderate and extreme environments. Although at lower taxonomic ranks, biogeographical patterns are apparent for some virus groups [146, 147], including such globally distributed viruses as crAssphage and its relatives [148], the distribution of viruses will depend on a number of virus- and host-specific traits [149], precluding or, at least, complicating generalizations. Overall, it appears that the non-uniform environmental distribution of viruses owes more to the biology of the host organisms that determine the host range than to physicochemical characteristics of the environments.

### Abundance and diversity of viruses in the biosphere and virus/microbe ratios in different environments

Viruses are often considered to be the most abundant biological entities on Earth [150, 151]. Widely cited estimates give the hyperastronomical number of about 10^31^ virus particles present on Earth at any given time and a characteristic virus:microbe ratio (VMR) of 10 or greater [152, 153]. However, recent analyses present a more complex picture in which virus abundance and diversity as well as VMR strongly depend on the environment [154]. The previous high VMR values have been estimated primarily by detection of extracellular virions with epifluorescence microscopy or flow cytometry, which are prone to both missing intracellular viruses (including lysogens) and erroneously reporting non-viral material as virus particles [155]. Apparently more solid estimates for DNA viruses have been recently obtained by quantification of VHGs simultaneously with host hallmark genes in metagenomes. With the caveat that only DNA viruses were included in this analysis, generally lower VMR values have been estimated, with the mean of about 2. The highest VMR values were observed in aquatic metagenomes, with a mean of 3–4, whereas the characteristic VMR values in animal-associated metagenomes were found to be much lower, with the mean close to 1 [154], consistent with previous estimates [85, 156]. Intermediate VMR values were reported for soils, sediments, and microbial mats. These findings are likely to result in a revision of the virus abundance in the biosphere toward slightly lower values (perhaps, down from ≈10^31^ to ≈10^30^) but nevertheless support the notion that there are substantially more viruses than cells both on the planetary scale and in most specific environments. Furthermore, VMR is not necessarily a linear function of the microbial density in an environment. Thus, analysis of thousands of estimates from various marine samples has shown that the VMR is best described by power law functions with exponents less than one, which implies that this ratio drops with increased microbial abundance [157]. In the case of temperate duplodnavirians, this dependence is at least in part explained by the piggyback-the-winner model, according to which lysogeny becomes the strategy of choice for a virus at high host density [158].

The compositions of viromes in different types of environments can drastically differ. Generally, duplodnavirian caudoviricetes are considered to be the most abundant group of viruses on Earth. However, the traditional approaches based on fluorescence microscopy appear to have been heavily biased toward these viruses versus the smaller particles of tailless, icosahedral viruses of the varidnavirian class *Tectiliviricetes*. Metagenomic estimates indicate that tectiliviricetes might even outnumber caudoviricetes in marine habitats [87], consistent with an electron microscopy analysis that suggested that non-tailed virions outnumber the tailed ones in marine samples [159]. In contrast, quantitative analysis of the human gut metagenomes indeed reveals a heavy dominance of caudoviricetes, whereas tectiliviricetes account only for ≈1% of viral DNA [89]. Abundance estimates for RNA and ssDNA viruses across environments are lacking because these viruses cannot be efficiently visualized by epifluorescence microscopy. Unfortunately, this challenge cannot be fully overcome using the VHG-based VMR estimations because metatranscriptomics cannot be systematically used to determine VMR values for RNA viruses, whereas for ssDNA viruses, only the replicative and integrated intracellular forms of viral genomes could be quantified. Nevertheless, a quantitative analysis of viral community DNA in offshore sediments showed that ssDNA viruses constituted 96%–99% of the benthic total DNA viral assemblages [160], suggesting that, similar to tectiliviricetes, ssDNA viruses might outnumber duplodnavirians in some environments. Given the recent realization that RNA viruses might represent a more prominent component of the bacterial virome than currently appreciated (see above), the abundance of RNA viruses in the environment might also be non-negligible. Indeed, estimation of the relative abundances of RNA and DNA viruses using a mass ratio approach, whereby the genome length-normalized mass of RNA and DNA content in the viral buoyant density range is compared, showed that RNA viruses could contribute up to 65% of the total virioplankton in Antarctic waters [161]. Future efforts should be directed at developing an all-encompassing methodology for estimating abundance of viruses regardless of their genome type and length.

Looking at the virosphere from a different angle, what is the overall diversity of viruses and viral genes in the biosphere? To obtain accurate numbers, far more complete sampling of the global virome is required, but simple ballpark estimates seem to be feasible and potentially instructive [61]. The greatest diversity of viruses on Earth is represented by bacterial duplodnavirians and varidnavirians, with the distinct viromes of archaea and eukaryotes being relatively small additions. It can be conservatively assumed that there are 10^6^–10^7^ species of bacteria in the biosphere (some estimates suggest an order of magnitude greater numbers or even more). Most of these bacteria are hosts to multiple viruses. For *Escherichia coli* alone, about 100 distinct viruses have been reported, whereas for *Mycobacterium smegmatis*, more than 2000 distinct virus genomes have been sequenced [162]; none of these numbers is likely to represent the entire virome of the respective bacterium. Assuming, again conservatively, 10–100 distinct viruses per bacterial host species, the global virome is estimated to contain 10^7^–10^9^ distinct viruses. The upper limit of this range appears to be more realistic, given the highly conservative assumptions. Obviously, with ≈11 300 virus species currently established [163], and even taking into account the recent expansion of the virosphere through metagenome and metatranscriptome mining, we are still quite far from a complete description of the virosphere.

These simple calculations can be extended to estimate the size of the global virus pangenome, i.e. the total number of distinct genes in the global virome [61]. In addition to VHGs and other, moderately conserved genes involved in viral genome replication and virion formation, genomes of large viruses in realms *Duplodnaviria* and *Varidnaviria* typically contain multiple lineage-specific genes that are being increasingly found to counteract the host antivirus defenses [164]. Assuming, once again conservatively, 10 such unique genes per distinct virus, we obtain a likely low bound of the global viral pangenome at a staggering 10^10^ genes.

## Concluding remarks

In the last decade, metagenomics and metatranscriptomics have transformed the study of virus diversity and hence have changed the state of virus research in general. Indeed, with these approaches, the viromes from a great variety of diverse habitats can be studied in their entirety without relying on the traditional methods of growing and isolating viruses by infecting cultivated hosts or their cells that are permissive to only a miniscule fraction of the global virome. The result is a more than an order-of-magnitude increase in the known diversity of viruses, including numerous new groups that are only distantly related to previously known ones. Some of these new groups are extremely abundant, as strikingly exemplified by crassvirals, the most common human-associated viruses that were completely unknown prior to the advent of advanced metagenomics. These advances stimulated the development of a comprehensive taxonomic framework for viruses, spanning the range of taxa from realms at the top to species at the bottom. In the three years elapsed since the adoption of this new virus taxonomy, numerous novel taxa, including small new realms, have been created and more are expected to emerge whereas some established major taxa appear to require rearrangement. In particular, three current vast realms seem to require splitting into two or more independent realms. Nevertheless, the general organizational contours of the virosphere seem to have been elucidated.

Concomitant with the major increase in virus diversity, the advances of metagenomics and metatranscriptomics uncover patterns of host range and global ecology. The viromes of the three domains of life differ drastically in their composition, and there are substantial non-uniformities in the distribution of viruses among their hosts within each of the domains. The distribution of viruses across ecosystems appears to be determined primarily by host range patterns, i.e. the biome that in itself is affected by the environmental factors shapes the virome. In addition, specific adaptations of virus proteins to abiotic factors have been discovered in particular in extreme geothermal and hypersaline environments. Viruses appear to be the most abundant entities on Earth, with the number of virus particles significantly exceeding the number of cells. Rough estimates of the size of the global virome point to about a billion distinct viruses, with at least an order of magnitude more unique genes, a huge, largely untapped reservoir of genetic diversity.

### Glossary

- **Megataxonomy:** classification of viruses into taxa at or above the order rank to reflect the established macroevolutionary relationships of large virus groups and the nomenclature for these megataxa
- **Monophyletic:** belonging to a group of organisms that consists of all the descendants from a common ancestor (antonym: polyphyletic)
**-viraen(s)**: virus(es) of kingdom -*virae*
- **Viral hallmark gene (VHG)**—gene or parts of a gene that is conserved in diverse groups of viruses; used to define megataxa
**-viral(s)**: virus(es) of order -*virales*
**-virian(s)**: virus(es) of realm -*viria*
**-viricete(s)**: virus(es) of class -*viricetes*
**-viricot(s)**: virus(es) of phylum -*viricota*
**-virid(s)**: virus(es) of family -*viridae*
- **Virome**: the totality of viruses in a given habitat or environment
- **Virosphere**: the totality of all viruses in the biosphere
**-virus(es)**—virus(es) of genus -*virus*

## Data Availability

Data sharing is not applicable to this article as no datasets were generated or analyzed during the current study.
